# Platelet mitochondria dysfunction in diabetes mellitus: mechanisms and therapeutic implications

**DOI:** 10.3389/fphar.2026.1774791

**Published:** 2026-03-27

**Authors:** Srikanth Yadava, Harikrishna Reddy Dontiboina, Sajusha Dugluri, Ganesh Yadagiri, Priyanka Choudhury, Ramakrishna Kakarla

**Affiliations:** 1 KL College of Pharmacy, Koneru Lakshmaiah Education Foundation, Guntur, Andhra Pradesh, India; 2 Norstella, Gurugram, Haryana, India; 3 Department of Pharmacology and Toxicology, National Institute of Pharmaceutical Education and Research, Kolkata (NIPER-K), Kolkata, West Bengal, India; 4 Children’s Research Institute, Medical College of Wisconsin, Milwaukee, WI, United States

**Keywords:** diabetes, platelets, platelet aggregation, platelet mitochondria, platelet energy, thrombosis, vascular complications

## Abstract

Platelets play a pivotal role in hemostasis, thrombosis, and inflammation, and their dysfunction in diabetes significantly contributes to vascular complications such as ischemic stroke, myocardial infarction, and peripheral artery disease. This review explores the mechanisms underlying platelet hyperactivity in diabetes, emphasizing the critical involvement of platelet mitochondria. Hyperglycemia, insulin resistance, oxidative stress, advanced glycation end products, calcium dysregulation, and protein kinase C activation all converge to impair platelet mitochondrial function, leading to increased reactive oxygen species, altered bioenergetics, and defective mitophagy. These changes promote a pro-thrombotic and pro-inflammatory state, exacerbating vascular injury. Furthermore, the review highlights emerging therapeutic strategies targeting platelet mitochondria, including pharmacological agents, mitochondrial antioxidants, and even mitochondrial transplantation, to restore platelet function and mitigate vascular risks in diabetic patients. Understanding the intricate relationship between platelet mitochondria and diabetes opens new avenues for preventing and treating diabetic vascular complications.

## 1 Introduction

Platelets, also known as thrombocytes, are small cytoplasmic anucleate fragments that circulate in the bloodstream and are derived from the cytoplasm of megakaryocytes in bone marrow through a process of proplatelet extension and fragmentation called thrombopoiesis (Knight et al., 2017). Traditionally, platelets are recognized as central mediators of thrombosis, and recent studies have underscored the potential of platelets' role in inflammation, immunity, central nervous system diseases, and intercellular communication, particularly in pathological conditions (Mandel et al., 2022). Platelets' ability to secrete bioactive molecules and interact with both vascular and immune cells makes them key contributors to physiological homeostasis as well as pathological dysfunction (Yan et al., 2024). Platelet dysfunction is known to contribute to several cardiovascular diseases, including atherosclerosis, myocardial infarction (MI), ischemic stroke (IS), peripheral artery disease (PAD), deep vein thrombosis (DVT), and other complications such as cancer and autoimmune disorders (AID), among others (Ramakrishna et al., 2022b; Tian et al., 2025). Moreover, metabolic diseases such as diabetes mellitus (DM), obesity, and dyslipidemia have dysfunctional platelet activity, thereby promoting a pro-thrombotic and pro-inflammatory environment, leading to vascular complications like IS (Guo et al., 2021; Schneider, 2009).

Diabetes mellitus is a major metabolic disorder (Runthala et al., 2025) that is strongly associated with both macrovascular and microvascular complications, largely driven by platelet dysfunction (Choudhury and Cai, 2025; Ferreiro and Angiolillo, 2011). Macrovascular manifestations include IS, MI, and PAD, while microvascular complications such as retinopathy, neuropathy, cardiomyopathy, and nephropathy are highly prevalent in patients with diabetes (Choudhury et al., 2024a; Kaur et al., 2018; Prakash et al., 2025). Beyond these clinical complications, diabetes is characterized by a sustained prothrombotic and proinflammatory milieu in which platelets play a central pathogenic role (Gauer et al., 2022). Platelet hyperactivity in diabetes arises from multiple interrelated mechanisms, including chronic hyperglycemia–induced protein glycation, insulin resistance, mitochondrial dysfunction, oxidative stress, dysregulated lipid metabolism, endothelial dysfunction, chronic inflammation, elevated thromboxane production, and increased release of immature platelets, collectively triggers thrombotic risk (Greaves and Pula, 2025). Platelet mitochondria have emerged as critical regulators of this hyperreactive phenotype. Mitochondrial dysfunction in platelets promotes excessive reactive oxygen species (ROS) generation, calcium (Ca^2+^) dysregulation, and impaired bioenergetics, which together amplify granule secretion and sensitize platelets to metabolic stress (Ajanel et al., 2023). These mitochondrial defects perpetuate platelet activation, aggregation, and thrombosis, thereby exacerbating vascular complications in diabetes (Kakouros et al., 2011). Integrating these insights with platelet hyperreactivity, mitochondrially compromised platelets acts as key contributors to microvascular thrombosis, inflammation, and disease progression of diabetic comorbidities. The present review summarize current evidence on platelet dysfunction in diabetes, with particular emphasis on the role of platelet mitochondrial dysfunction in driving platelet hyperactivation, aggregation, and thrombosis. Additionally, this review explores emerging therapeutic strategies targeting platelet mitochondria as a potential approach to mitigate macrovascular and microvascular complications in the diabetic population.

## 2 Platelet physiology in health and diabetes

Despite the absence of a nucleus, platelet function is regulated by the organization and coordinated activity of its intracellular organelles, particularly mitochondria and secretory granules (Melchinger et al., 2019). The interplay between these organelles regulates platelet bioenergetics, Ca^2+^ homeostasis, and activation-dependent secretion. Disruption of these processes leads to platelet dysfunction and/or hyper-reactivity. Platelets consist of a variety of granules, including dense granules, alpha granules (α-granules), lysosomes, and microvesicles (George et al., 2018). Collectively, these serve as critical reservoirs of bioactive molecules that regulate platelet activation, aggregation, inflammation, platelet apoptosis, and vascular homeostasis, underscoring the functional complexity of platelets despite their anucleate nature (Kakarla et al., 2024). The functions of various platelet components are discussed below.

### 2.1 Platelet mitochondria

Platelets consist of a lower number of mitochondria (4–8) and contain other cell organelles including dense tubular system, Golgi apparatus, lysosomes, ribosomes, glycogen granules, alpha granules, and dense granules. These organelles in platelets, in coordination with cellular DNA, synthesize various proteins which regulates ATP production, ROS generation, Ca^2+^ balance, and apoptosis (Melchinger et al., 2019). Although platelets contain a low number of mitochondria, they are highly important for platelets to maintain the normal flow of the blood (Ajanel et al., 2023). Mitochondria constantly provide high energy demand in platelets to maintain their normal function (George et al., 2018). However, following vascular injury, platelet mitochondria become highly active and provide the required ATP to meet the platelet needs (Kulkarni et al., 2022). Loss of mitochondrial membrane potential (ΔΨM), opening of mitochondrial permeability transition pore (mPTP), release of cytochrome C, Ca^2+^ imbalances, alterations in mitochondrial bioenergetics, mitophagy, and dynamic changes in mitochondrial number have been reported in the platelet mitochondria in DM (Wu et al., 2015).

### 2.2 Alpha and dense granules

Alpha granules (AGs) are the largest and most abundant platelet granules (∼40–80), containing over 300 bioactive proteins essential for hemostasis, inflammation, and tissue repair (Italiano and Battinelli, 2009). Adhesion proteins (fibrinogen, vWF, fibronectin, thrombospondin), clotting factors (V, XI, XIII, prothrombin, protein S), growth factors (PDGFs, TGF-β, IGF-1, VEGF, EGF), and cytokines/chemokines (PF4/CXCL4, CCL5, IL-8) are the major components of AGs that mediate platelet activation, vascular repair, and immune signaling (Ambrosio and Di Pietro, 2025; Blair and Flaumenhaft, 2009). Furthermore, AG has surface proteins like P-selectin and αIIbβ3, which facilitate platelet-leukocyte/endothelial interactions. Apart from these platelet factors, AGs also store amyloid precursor protein (APP) and amyloid-β (Aβ) (Kucheryavykh et al., 2017).

Dense granules (DGs), also referred to as δ-granules, are smaller in number (3–8) and in size compared to AGs. These granules are often the first and rapidly released granules upon initial platelet adhesion. DGs contain the platelet activation amplifying compounds that are crucial for maintaining the platelet aggregation, as well as recruiting immune and vascular cells (Sharda and Flaumenhaft, 2018). DGs are non-proteinaceous storage vesicles that contain ADP and ATP in a ratio of 2:3 (amplifying platelet activation) (Dupuis et al., 2020), Ca^2+^ ions (platelet aggregation and clot formation), and serotonin (vasoconstriction) (Rawish et al., 2020). High metabolic stress induced by diabetes in platelet mitochondria elevates ROS, disrupts Ca^2+^ buffering, and enhances ADP release via excessive degranulation of DGs, which significantly amplifies the P2Y12-Gi-IP_3_ loops driving platelet hyperreactivity (Hu et al., 2017). The contents in AGs and DGs are illustrated in Table 1.

**TABLE 1:** Various types of platelet granules and their contents with physiological functions.

Platelet granules	Morphology of granules	Contents of granules	Physiological roles	References
Alpha (α) granules	• Largest (∼200–250 nm) and most abundant platelet granules (∼40–80) • Protein-rich, membrane-bound vesicles	Adhesion proteins: fibrinogen, vWF, fibronectin, thrombospondin	• Platelet adhesion to exposed subendothelium • Platelet–platelet cohesion • Stabilization of the primary hemostatic plug	Blair and Flaumenhaft (2009)
		Coagulation factors Factor V, XI, XIII, prothrombin	• Localized thrombin generation • Fibrin clot stabilization	Blair and Flaumenhaft (2009), Harrison and Cramer (1993)
		Anticoagulant/regulatory proteins: Protein S, protease inhibitors	• Regulation of clot size • Prevention of excessive thrombosis	Harrison and Cramer (1993), Kucheryavykh et al. (2017)
		Growth factors: PDGF (aa, ab, bb), TGF-β1, TGF-β2, IGF-1, VEGF, EGF	• Tissue repair and wound healing • Cellular proliferation • Angiogenesis following vascular injury	Blair and Flaumenhaft (2009), Cecerska-Heryć et al. (2022)
		Cytokines and chemokines: PF4/CXCL4, CCL5 (RANTES), IL-8 (CXCL8)	• Leukocyte recruitment • Platelet-mediated inflammatory and immune signaling	Ambrosio and Di Pietro (2025), Herter et al. (2014)
		Surface proteins P-selectin, CD63, integrin αIIbβ3 (GPIIb/IIIa)	• Platelet–leukocyte aggregation • Platelet–endothelial interaction • Enhanced platelet activation	Ambrosio and Di Pietro (2025), Blair and Flaumenhaft (2009)
		Amyloid-related proteins: amyloid precursor protein (APP), amyloid-β (Aβ)	• Platelets as a peripheral source of amyloid proteins • Contribution to vascular and thromboinflammatory pathology	Kucheryavykh et al. (2017)
Dense (δ) granules	• Small (∼150 nm), electron-dense vesicles • Non-proteinaceous storage organelles, High calcium content	ADP and ATP (2:3 ratio)	• Amplification of platelet activation • Sustained platelet aggregation and recruitment	Dupuis et al. (2020), Sharda and Flaumenhaft (2018)
		Ca^2+^	• Platelet shape change • Activation of aggregation and coagulation pathways	Sharda and Flaumenhaft (2018)
		Serotonin (5-HT)	• Vasoconstriction at injury sites • promotes platelet aggregation	Rawish et al. (2020)
Platelet lysosomes	• Acidic, membrane-bound vesicles • Classical lysosomal morphology	Acid hydrolases	• Degradation of internalized and extracellular substrates • Tissue remodeling	Heijnen and van der Sluijs (2015)
		Acid phosphatase	• Breakdown of phosphate-containing macromolecules	Heijnen and van der Sluijs (2015)
		Arylsulfatases	• Degradation of sulfated substrates	Heijnen and van der Sluijs (2015)
		β-hexosaminidase	• Degradation of glycosaminoglycans	Heijnen and van der Sluijs (2015)
		β-glucuronidase	• Cleavage of glucuronic acid residues	Heijnen and van der Sluijs (2015)
		Cathepsins B, L, D	• Proteolysis of extracellular matrix components • Support tissue remodeling and inflammation	Heijnen and van der Sluijs (2015)
		LAMP-1 and LAMP-2	• Protection of lysosomal membrane • Markers of platelet activation	Bouhamdani et al. (2021)
		Synergistic interaction with α-granule factors (PDGF, TGF-β, VEGF)	• Enhanced tissue repair • Angiogenesis	Burnouf et al. (2023), Ren et al. (2008)
Platelet-derived microvesicles (PMPs)	• 100–1,000 nm membrane vesicles • Shed upon platelet activation • Externalized phosphatidylserine (PS)	PS	• Highly procoagulant surface • Assembly of coagulation complexes	Vajen et al. (2015), Zaldivia et al. (2017)
		Membrane proteins: GPIb, GPIIb/IIIa (αIIbβ3), P-selectin	• Platelet adhesion • Interaction with leukocytes and endothelial cells	Lazar and Goldfinger (2021), Zaldivia et al. (2017)
		Granule-derived cargo: proteins, cytokines, ADP	• Amplification of coagulation • Promotion of inflammation	Vajen et al. (2015)
		RNA and microRNAs	• Modulation of gene expression in recipient cells	Puricelli et al. (2023)
		Mitochondrial fragments	• Induction of inflammatory and oxidative stress responses	Puricelli et al. (2023)
		Bioactive lipids and proteins	• Intercellular transfer of prothrombotic and inflammatory signals	Vajen et al. (2015)

Under normal physiological conditions, vascular injury initiates platelet tethering via GPIb-IX-V binding to vWF under shear, followed by firm adhesion and activation through GPVI engagement of exposed collagen, which triggers downstream intracellular signaling cascades (Varga-Szabo et al., 2008; Zhang et al., 2022). This activation promotes ADP and thromboxane A_2_ (TXA_2_)-mediated amplification and thrombin-dependent inositol 1,4,5-trisphosphate receptor (IP_3_R)-driven Ca^2+^ release from dense tubular stores (DTS), resulting in a transient rise in cytosolic Ca^2+^(Jardin et al., 2007; Millington-Burgess and Harper, 2021). Under physiological conditions, mitochondrial Ca^2+^ uptake via the mitochondrial calcium uniporter (MCU) buffers cytosolic Ca^2+^ rises during platelet activation, preserving ΔΨm and preventing opening of the mPTP (Lonobile et al., 2025; Millington-Burgess and Harper, 2021). This controlled mitochondrial handling of Ca^2+^ supports metabolic output and enables soluble N-ethylmaleimide-sensitive factor attachment protein receptors (SNARE) and their proteins (syntaxin-2/11, SNAP-23, VAMP-7/8) mediated graded α-granule exocytosis necessary for platelet plug stabilization without producing excess thrombus (Pokrovskaya et al., 2018). Similar to AGs, DGs release is facilitated by platelet mitochondrial ATP as well as the cytosolic Ca^2+^signaling. Rapid Ca^2+^ rise via IP_3_R/TPC2 stores and redox-sensitive PKC signaling facilitates SNARE fusion and granule exocytosis (Ambrosio et al., 2015). Chronic hyperglycemia increases polyol pathway flux via aldose reductase, depleting NADPH and promoting mitochondrial ROS accumulation. Oxidative stress enhances p53 Ser15 phosphorylation and mitochondrial stress signaling, destabilizing Bcl-xL-mediated preservation of ΔΨm and ATP production (Tang et al., 2014). Concurrent ROS and Ca^2+^ dysregulation promote excessive MCU-dependent mitochondrial Ca^2+^ uptake, lowering the threshold for mPTP opening and amplifying phosphatidylserine (PS) exposure and procoagulant activity (Choo et al., 2012). Collectively, AGs and DGs link hemostasis, immunity, and vascular inflammation, with increased relevance in diabetic mitochondrial dysfunction and thromboinflammatory pathologies (Blair and Flaumenhaft, 2009; Melchinger et al., 2019; Sagar et al., 2022).

### 2.3 Platelet lysosomes

Platelet lysosomes contain various enzymes, including acid hydrolases (degradation), acid phosphatase (breakdown of phosphate-containing macromolecules), arylsulfatases (sulfated substrate degradation), β-hexosaminidase (degrades glycosaminoglycans), β-glucuronidase (cleaves glucuronic acid residues), and cathepsins B, L, and D (proteolysis) (Heijnen and van der Sluijs, 2015). Along with these, the lysosomal membrane contains lysosome-associated membrane proteins (LAMP-1 and LAMP-2), considered as markers for platelet activation that prevent hydrolases from digesting the lysosomal membrane (Bouhamdani et al., 2021). Moreover, lysosomal enzymes work synergistically with alpha granule bioactive substances like PDGF, TGF-β, and VEGF for tissue repair and angiogenesis (Burnouf et al., 2023; Ren et al., 2008). Compared with alpha and dense-granule release, lysosomal discharge is limited and requires strong activation (such as thrombin), contributing to localized proteolysis and remodeling within the developing thrombus (Lee et al., 2016).

Importantly, lysosomal activity in platelets is functionally integrated with mitochondrial homeostasis. Despite lacking a nucleus, platelets retain functional autophagic machinery that enables the clearance of damaged mitochondria (Schwertz and Middleton, 2023). Loss of ΔΨM and increased ROS from the mitochondria act as signals for the targeting of dysfunctional mitochondria to lysosomes for degradation. This process requires adequate lysosomal acidification through V-ATPase activity, which depends on ATP predominantly generated by mitochondria (Schwertz and Middleton, 2023). Consequently, mitochondrial bioenergetic impairment can compromise lysosomal function and reduce mitophagic efficiency (Wu et al., 2024). Elevated mitochondrial ROS production in diabetes promotes mitochondrial damage, followed by impaired lysosomal autophagy and subsequent accumulation of damaged mitochondria. Evidence of impaired autophagic flux in diabetic platelets further supports disruption of mitochondrial quality control (Wu et al., 2024). Overall, disruption of mitochondrial-lysosomal coordination in diabetes can result in sustained oxidative stress and promote platelet hyperreactivity, thereby contributing to thrombotic risk and vascular complications (Lee et al., 2016).

### 2.4 Platelet-derived microparticle/Microvesicles

During platelet activation, platelets produce various particles called platelet-derived microparticles/microvesicles (PMPs). Platelet microvesicles display PS on their outer surface and carry several platelet membrane proteins, including glycoprotein Ib (GPIb), glycoprotein IIb/IIIa (GPIIb/IIIa/αIIbβ3), and P-selectin (Lazar and Goldfinger, 2021; Zaldivia et al., 2017). PMPs also encapsulate cargo derived from alpha granules and dense granules, such as proteins, cytokines, and ADP. In addition, microvesicles contain RNA and microRNAs and may even transport small amounts of mitochondria or mitochondrial fragments, contributing to their potent biological activity (Puricelli et al., 2023). PMPs are formed because of platelet activation, high shear stress in blood vessels, oxidative stress, mitochondrial dysfunction, and apoptosis-like changes. Indeed, PMPs are highly procoagulant and involved in platelet aggregation, inducing inflammation, transferring lipids, proteins, microRNAs, and mitochondrial fragments, eventually contributing to the thrombotic events (Vajen et al., 2015). Diabetic platelets show amplified platelet derived microvesicles (PMVs) release as a result of excessive oxidative stress, further enhancing coagulation and vascular inflammation. Elevated circulating PMVs in diabetic patients are therefore considered both markers and mediators of thrombotic risk (Bergen et al., 2020; Vajen et al., 2015).

### 2.5 Platelet receptors

The external membrane of platelets serves as a specialized dynamic interface that enables the complex interactions between platelets and damaged vasculature/nearby platelets/leucocytes through receptors (O'Donnell et al., 2014; Ramakrishna et al., 2022a). The external membrane comprises a complex phospholipid bilayer, which is composed of scattered cholesterol, sphingolipids, and unsaturated acyl acids (Komatsuya et al., 2020). Several receptors, such as transmembrane glycoproteins, integrins, immunoglobulin (Ig) superfamily receptors, and G-protein-coupled receptors (GPCRs), are embedded within the platelet outer membrane matrix for the purpose of mediating platelet activation, adhesion, aggregation, and signal transduction during thrombosis (Bodin et al., 2003). Based on biological function platelet receptors can be classified into several types including adhesion receptors (Glycoprotein Ib-IX-V (GPIb-IX-V), glycoprotein VI (GPVI), integrins such as integrin alpha IIb beta 3 (αIIbβ3), activation receptors including protease-activated receptors (PARs), purinergic receptors P2Y1 and P2Y12 (P2Y1/P2Y12), and thromboxane prostanoid receptors (TP receptors), regulatory and inhibitory receptors such as platelet endothelial cell adhesion molecule-1 (PECAM-1/CD31) and platelet glycoprotein IV (GPIV/CD36), immunological receptors including Fc gamma receptor IIA (FcγRIIA) and Toll-like receptors (TLRs), and other specialized receptors such as C-type lectin-like receptor 2 (CLEC-2) and thrombopoietin receptors (TPO receptors) (Jackson, 2007).

Platelet activation begins with adhesion receptors that sense vascular injury. The GPIb-IX-V complex is the primary receptor for vWF under high shear flow. This complex consists of GPIbα, GPIbβ, GPIX, and GPV, with GPIbα acting as the major vWF-binding subunit (Zhang et al., 2022). Engagement of GPIbα with vWF triggers mechanosensitive signaling, recruiting adaptor proteins such as 14-three to three and filamin, and activating Src family kinases (Lyn, c-Src). This leads to phosphorylation of guanine nucleotide exchange factors (Vav), activation of Rac1, cytoskeletal reorganization, and phosphoinositide 3-kinase (PI3K)-protein kinase B (PKB/AKT) pathway and mitogen-activated protein kinase (MAPK) signaling. GPIb-IX-V also activates phospholipase C gamma 2 (PLCγ2), initiating IP_3_-diacylglycerol (DAG) signaling, intracellular Ca^2+^ release, and granule secretion. Defects in this complex result in Bernard-Soulier syndrome, characterized by macrothrombocytopenia and mucocutaneous bleeding (Zhang et al., 2022). Following initial tethering, platelets bind to exposed collagen through the GPVI-FcRγ complex, a key mediator of firm adhesion and activation. GPVI ligation leads to phosphorylation of immunoreceptor tyrosine-based activation motifs (ITAMs) on the FcRγ chain, activating Syk, PLCγ2, and downstream IP_3_-DAG signaling for Ca^2+^ mobilization (Fuentes, 2022; Gibbins et al., 1997). Although collagen is the primary ligand, GPVI also interacts with fibrin, laminin, fibronectin, vitronectin, galectin-3, adiponectin, and EMMPRIN, linking it to thrombus stabilization and vascular inflammation (Moroi et al., 2021). Despite its central role in platelet activation, GPVI deficiency results in only mild bleeding, making it an attractive antithrombotic target (Perrella et al., 2021).

Integrin αIIbβ3 (GPIIb/IIIa) is the most abundant platelet integrin, binds with fibrinogen and vWF to bridge adjacent platelets. Its activation requires inside-out signaling, in which agonists such as thrombin, ADP, and thromboxane A_2_ (TXA_2_) activate PLC to generate IP_3_ and DAG, raising intracellular Ca^2+^ and activating Ca^2+^ and DAG-GTP Exchange Factor I (CalDAG-GEFI). This promotes Ras-related protein 1 (Rap1) activation, recruitment of Rap1-GTP-interacting adaptor molecule (RIAM), and binding of talin and kindlin-3 to β3, switching the integrin into a high-affinity state (Huang et al., 2019). Further, binding of ligands such as fibrinogen and vWF then initiates outside-in signaling, involving integrin clustering, Gα13, Src family kinases, Syk, and downstream adaptors (SLP-76, Vav, PLCγ2), driving spreading, cytoskeletal remodeling, and granule secretion (Durrant et al., 2017). Integrin α2β1 (glycoprotein Ia/IIa (GPIa/IIa)) contributes to stable adhesion to collagen by recognizing GFOGER motifs in the collagen triple helix. Unlike GPVI, α2β1 primarily functions as an anchoring receptor under physiological shear but cooperates with other integrins through inside-out and outside-in signaling (Lemmens et al., 2024). Loss of α2β1 delays, but does not prevent, platelet adhesion, though it substantially contributes to arterial thrombosis. Other platelet integrins, including α5β1 (fibronectin receptor), α6β1 (laminin receptor), and αvβ3 (vitronectin receptor), play supportive roles in adhesion (Janus-Bell and Mangin, 2023).

Further, platelet activation is amplified by GPCRs. P2Y1 (Gq-coupled) generates Ca^2^-mediated shape change, while P2Y12 (Gi-coupled) sustains aggregation by inhibiting cAMP and activating PI3K-Akt and integrins (Patel et al., 2025). P2X1, a ligand-gated ion channel activated by ATP, triggers rapid Ca^2+^ influx, contributing to early activation (Koupenova and Ravid, 2018). Thrombin receptors, like protease-activated receptors (PAR)1 and PAR4, signal through Gq, Gi, and G12/13 pathways. PAR1 initiates rapid activation, while PAR4 provides sustained signaling (French and Hamilton, 2016). TP receptors, activated by TXA_2_, couple to Gq and G12/13 to enhance integrin activation and promote vasoconstriction and inflammation (Eckenstaler and Benndorf, 2025). Collectively, these receptors coordinate platelet adhesion, activation, and aggregation to form a stable hemostatic plug (Tian et al., 2025).

Indeed, platelet mitochondria and receptor mechanisms are cohesively interconnected. Engagement of GPVI, PARs, and purinergic receptors activates PLC and increases intracellular Ca^2+^, part of which is taken up by mitochondria via the MCU. This initially enhances oxidative phosphorylation (OXPHOS) to meet energetic demands (Bergen et al., 2020; Grichine et al., 2023). However, sustained Ca^2+^ promotes partial loss of ΔΨm, and exaggerates ROS generation (Siewiera et al., 2022). Released ROS acts as a secondary signaling amplifier by inhibiting redox-sensitive protein tyrosine phosphatases and sustaining Src and Syk phosphorylation downstream of GPVI and integrins. Moreover, ROS also enhances PI3K-Akt signaling and facilitates Rap1-dependent inside-out activation of αIIbβ3, lowering the threshold for stable aggregation (Carrim et al., 2014). Concurrent mitochondrial depolarization further disrupts Ca^2+^ handling and promotes PS exposure, increasing procoagulant activity. This suggests that metabolic dysregulation of platelet mitochondria in hyperglycemia may produce exaggerated Ca^2+^ mobilization and amplified downstream signaling by lowering the receptor threshold, leading to a prothrombotic state (Siewiera et al., 2022). Overall, various platelet receptors work together to make a initial platelet plug, later which turns into stable clot.

### 2.6 Emerging platelet receptors

Unregulated platelet activation leads to a severe risk of thrombus aggregation and pathological thrombosis; therefore, platelet activation is tightly regulated by inhibitory and regulatory receptors that act as endogenous checkpoints, helping to prevent inappropriate activation and maintain hemostatic balance (Bye et al., 2016). Unlike platelet activation receptors that promote Ca^2+^ mobilization and integrin activation, inhibitory receptors act by mechanisms such as cyclic nucleotide elevation (cAMP, and cGMP) and immunoreceptor tyrosine-based inhibition motif (ITIM)-mediated phosphatase recruitment to suppress pro-aggregatory signaling (Bye et al., 2016; Scridon, 2022). In addition to well-characterized platelet agonists such as collagen, thrombin, ADP, and thromboxane A_2_, several emerging or less conventional receptors have gained attention for their roles in modulating platelet activation under pathological or inflammatory conditions. These receptors are not primary mediators of hemostasis but become particularly relevant during immune-thrombotic responses, sepsis, or sterile inflammation, where they contribute to dysregulated platelet reactivity and thrombosis. They include the prostacyclin receptor (IP receptor) for cAMP signaling (Rogula et al., 2021), nitric oxide (NO) soluble guanylate cyclase (sGC) pathway for cGMP responses (Balmes et al., 2024), adenosine receptors (Boncler et al., 2024), G6b-B (Soriano Jerez et al., 2021), carcinoembryonic antigen-related cell adhesion molecule 1/2 (CEACAM-1/2) (Wong et al., 2009), leukocyte-associated immunoglobulin-like receptor-1 (LAIR-1) (Smith et al., 2024), FcγRIIb, Sialic acid binding Ig like lectins (Siglecs), and ITIM-bearing receptors such as PECAM-1-1 (Smith et al., 2024) and G6b-B that recruit phosphatases (Soriano Jerez et al., 2021). Future studies need to underscore the therapeutic potential of these receptors in combating platelet activation, aggregation, and thrombosis. Table 2 summarizes various platelet receptors and their role in activation and aggregation.

**TABLE 2:** Platelet receptors and their role in platelet activation and aggregation.

Receptor	Receptor class and signaling	Primary ligand(s)	Copy number per platelet	Functional role	Reference(s)
GPIb-IX-V complex	Transmembrane glycoprotein; mechanosensitive Src–PI3K–PLCγ2 signaling	von Willebrand factor (vWF)	∼25,000	Activator: platelet tethering and adhesion under high shear (hemostasis, arterial thrombosis)	O'Donnell et al. (2014), Sivaraman and Latour (2011), Zhang et al. (2022)
GPVI–FcRγ complex	Ig superfamily with ITAM adaptor; Syk–PLCγ2 signaling	Collagen (primary); fibrin; laminin; fibronectin; vitronectin; galectin-3; adiponectin; EMMPRIN	∼4,000-6,000	Activator: collagen-induced activation, thrombus stabilization, vascular inflammation	Fuentes (2022), Gibbins et al. (1997), Moroi et al. (2021), Stegner et al. (2014)
Integrin αIIbβ3 (GPIIb/IIIa)	Integrin; inside-out and outside-in signaling via Rap1, talin/kindlin-3, Src/Syk	Fibrinogen; vWF	∼50,000-100,000	Activator: platelet aggregation and thrombus growth	Huang et al. (2019), Durrant et al. (2017), Lavergne et al. (2017)
Integrin α2β1 (GPIa/IIa)	Integrin; cooperative adhesion signaling	Collagen (GFOGER motifs)	∼2,000-4,000	Activator: stable platelet adhesion under physiological shear	Cosemans et al. (2008), Lemmens et al. (2024)
Integrin α5β1	Integrin: supportive adhesion signaling	Fibronectin	∼1900	Activator (supportive); adhesion reinforcement	Janus-Bell and Mangin (2023)
Integrin α6β1	Integrin: supportive adhesion signaling	Laminin	∼20,000	Activator, (supportive); adhesion reinforcement	Janus-Bell and Mangin (2023)
Integrin αvβ3	Integrin: low-abundance outside-in signaling	Vitronectin	∼50-100	Activator (supportive); thrombus stabilization	Janus-Bell and Mangin (2023), Saboor et al. (2013)
P2Y1	GPCR (Gq-coupled); PLC-Ca^2+^ signaling	ADP	∼150-300	Activator: early platelet shape change and activation	Pan et al. (2024), Patel et al. (2025)
P2Y12	GPCR (Gi-coupled); cAMP inhibition, PI3K–Akt signaling	ADP	∼400-1,000	Activator (amplification); sustained aggregation, clinical antiplatelet target	Mansour et al. (2020), Patel et al. (2025)
P2X1	Ligand-gated ion channel; Ca^2+^ influx	ATP	∼1,400	Activator rapid activation under high shear	Burkhart et al. (2012), Koupenova and Ravid (2018)
PAR1	GPCR (protease-activated); Gq/Gi/G12/13 signaling	Thrombin	∼1,500-2,500	Activator: rapid thrombin-mediated activation	Clemetson and Clemetson (2019), French and Hamilton (2016)
PAR4	GPCR (protease-activated); sustained signaling	Thrombin	∼500-1,000	Activator, prolonged thrombin responses	French and Hamilton (2016), Li S et al. (2021)
TP receptor	GPCR; Gq/G12/13 signaling	Thromboxane A_2_	∼1,000-1,500	Activator, amplification of aggregation and vasoconstriction	Eckenstaler and Benndorf (2025), Rand and Israels (2018)
FcγRIIA	Ig receptor with ITAM; Syk signaling	IgG immune complexes	∼1,000-5,000	Activator, immune-mediated platelet activation (immunothrombosis)	Jackson (2007), Zhang et al. (2024)
CLEC-2	C-type lectin-like receptor with hemITAM; Syk signaling	Podoplanin (canonical)	∼2,000-3,000	Activator: thrombo-inflammatory signaling	Gitz et al. (2014), Jackson (2007)
TLT-1	Ig superfamily (TREM family); no ITAM/ITIM, signals indirectly via platelet activation pathways	Ligand not definitively identified	∼ 5,000-50,000	Activator, stabilizes platelet aggregation; promotes platelet-leukocyte interactions; mediator of thromboinflammation	Washington et al. (2004)
TLRs (TLR2/4)	Pattern-recognition receptors; MyD88-dependent signaling	PAMPs/DAMPs	Low abundance	Regulatory; inflammation-associated platelet activation	Jackson (2007)
PECAM-1 (CD31)	Ig superfamily with ITIM; SHP-1/2 recruitment	Homophilic interactions	∼5,000-10,000	Inhibitory; endogenous suppression of platelet activation	Bye et al. (2016), Metzelaar et al. (1991), Smith et al. (2024)
GPIV (CD36)	Scavenger receptor; inflammatory modulation	Oxidized lipids	∼10,000-25,000	Regulatory; inflammation-linked platelet responses	Jackson (2007), Nergiz-Unal et al. (2011)
FcγRIIb	Ig receptor with ITIM	IgG immune complexes	-	Inhibitory; immune checkpoint	Bye et al. (2016)
G6b-B	ITIM-bearing receptor; phosphatase recruitment	Not fully defined	∼13,700- 25,000	Inhibitory; platelet homeostatic checkpoint	Coxon et al. (2012), Soriano Jerez et al. (2021)
CEACAM-1/−2	Ig superfamily with ITIM	Homophilic interactions	Low abundance	Inhibitory; immune-thrombotic regulation	Wong et al. (2009)
LAIR-1	Ig-like inhibitory receptor; ITIM signaling	Collagen-related ligands	-	Inhibitory; collagen-linked immune regulation	Smith et al. (2024)
Siglecs	Sialic acid-binding Ig-like lectins; ITIM signaling	Sialylated glycans	Low abundance	Inhibitory; immune-inflammatory modulation	She et al. (2025)
IP receptor	GPCR; cAMP signaling	Prostacyclin (PGI_2_)	Low abundance	Inhibitory; endothelial-derived platelet suppression	Rogula et al. (2021)
NO–sGC pathway	Enzyme-linked signaling; cGMP pathway	Nitric oxide	-	Inhibitory; endothelial platelet inhibition	Balmes et al. (2024)
Adenosine receptors	GPCR; cAMP signaling	Adenosine	Low abundance	Inhibitory; metabolic regulation of platelet activation	Boncler et al. (2024)

## 3 Platelet dysfunction and prothrombotic state in diabetes mellitus

Under physiological conditions, platelets operate at a relatively low metabolic rate. However, vascular injury swiftly alters this state into a high metabolic state to prevent blood loss. Activated platelets exhibit elevated ATP levels, indicating increased mitochondrial energy production driven by glycolysis, OXPHOS, and fatty-acid catabolism. Indeed, glucose is readily available for platelets to maintain physiological or pathological roles (vascular injury) (Fidler et al., 2017a). Glucose uptake by platelets is facilitated by glucose transporters (GLUT) like, GLUT-1 and GLUT-3. GLUT-1 is present on the platelet plasma membrane, whereas GLUT-3 is abundantly located in α-granules, and a low proportion is expressed on the plasma membrane under resting conditions (Fidler et al., 2017b). Interestingly, upon vascular injury, during platelet activation, GLUT-3 translocates to the plasma membrane, resulting in increased glucose uptake and enhanced ATP production (Fidler et al., 2017a). Overall, these findings suggested that elevated glucose uptake by platelets following vascular injury might contribute to further activation of platelets, platelet aggregation, and eventually thrombosis.

DM is associated with platelet hyper-responsiveness, which shifts the platelet hemostasis to a hypercoagulable, prothrombotic state with increased risk of thrombosis. Platelet hypersensitivity triggers the elevation of pro-aggregatory factors such as P-Selectin, vWF, decreases the membrane fluidity, and decreases the anti-aggregatory factors (Kakouros et al., 2011). On the other side, coagulation factors such as tissue factor (TF), factor VII, XII, XI, IX, and tissue factor pathway inhibitor (TFPI) are elevated along with the rise of fibrinogen and fibrin, prothrombin, and thrombin (Sobczak and Stewart, 2019). In Contrast, the fibrinolytics, which encounter the formed clots such as plasminogen, tissue-type plasminogen activator (tPA), plasminogen activator inhibitor-1 (PAI-1), and thrombin-activatable fibrinolysis inhibitor (TAFI) are downregulated in DM (Carrizzo et al., 2018; Li X et al., 2021). Together, platelet hypersensitivity, elevated coagulant factors, and decreased endogenous fibrinolytics induces the pro-thrombotic state in DM. Apart from these factors, comorbid conditions associated with DM, such as obesity (Samuels et al., 2019), dyslipidemia (Podrez et al., 2007), and non-alcoholic fatty liver disease (NAFLD) also promote the prothrombotic state in DM (Ciavarella et al., 2021).

Platelet hypersensitivity is characterized by increased platelet microparticles, ROS, platelet activation complex-1 (PAC-1) binding, P-selectin, thromboxane B2 (TXB2), and enhanced platelet spreading, number, mean platelet volume (MPV), and high aggregation to low concentrations of agonists. Further, it has been identified that platelet counts are elevated by 10% more in DM patients (both T1DM and T2DM). Moreover, MPV is also slightly elevated in a pre-diabetic state, suggesting that platelets are crucial in elucidating the cardiovascular risks in DM patients. Various factors, such as NO, prostaglandin I_2_ (PGI_2_), and insulin, act as antiaggregatory agents. However, high glucose alters the physiological actions of the above-mentioned agents, leading to platelet aggregation (Li X et al., 2021). Chronic hyperglycemia is reported to have increased expression of adhesion molecules and PAR4, which eventually release activated PMPs, thereby triggering production and secretion of interleukin-6 (IL-6), a pro-inflammatory and pro-thrombotic factor in DM. In addition, glycation of platelet membrane proteins, lowers the platelet membrane fluidity, which allows Ca^2+^ influx, subsequently promotes platelet activation, and aggregation (Giannella et al., 2021; Kaur et al., 2018; Schneider, 2009). Several mechanisms have been identified for the hypersensitivity of platelets in DM, including insulin resistance, oxidative stress, formation of advanced glycation end products (AGEs), Ca^2+^ dysregulation, and protein kinase C (PKC) activation, among others (Kaur et al., 2018). The detailed mechanisms responsible for platelet hypersensitivity in DM are discussed below.

### 3.1 Insulin resistance and signaling defects

Platelets possess insulin receptors (IRs) act as inhibitors of platelet activation through specific signaling pathways under normal physiological conditions. When insulin/insulin-like growth factor-1 (IGF-1) binds to its receptor on the platelet surface, it triggers a cascade involving insulin receptor substrate (IRS) proteins and leads to phosphorylation of protein kinase B (PKB/Akt) at Ser473 (Hunter and Hers, 2009; Randriamboavonjy and Fleming, 2009). This leads to an increase in intracellular cAMP concentrations and activates protein kinase A (PKA), ultimately suppressing platelet activation. This insulin signaling directly opposes the ADP receptor P2Y12, which, upon activation, inhibits adenylyl cyclase, which decreases cAMP levels and mitigates the PKA activation. Reduced cAMP and inactive PKA result in increased Ca^2+^ mobilization inside the platelet, facilitating activation and aggregation. Thus, the insulin receptor’s signaling pathway physiologically opposes the pro-aggregatory signals mediated by P2Y12 (Anja et al., 2012; Cattaneo, 2011). In a diabetic insulin-resistant state, platelets lose insulin-induced protein kinase B (PKB)-Ser473 phosphorylation, and the ability of insulin/IGF-1 to interfere with P2Y12 signaling to cAMP-dependent PKA is reduced (Ferreira et al., 2006). This failure of inhibitory insulin signaling leads to unregulated platelet activation and aggregation, resulting in hyperactive platelets and increased thrombotic risk (Randriamboavonjy & I. Fleming, 2009). Furthermore, experimental studies revealed that platelet P2Y12 receptor expression is fourfold higher in T2DM patients compared to healthy individuals (Hu et al., 2017). Overall, insulin resistance in DM significanly promoting the platelet activation, thereby thrombosis.

### 3.2 Advanced glycation end products

Advanced glycation end products (AGEs) accumulate extensively in diabetic vasculature and directly activate platelets through multiple mechanisms. AGEs interact with platelet GPIV receptors along with receptor for advanced glycation end products (RAGE) expressed on platelets, triggering nuclear factor-κB (NF-κB) activation and upregulating pro-inflammatory genes (Recabarren-Leiva et al., 2021; Zhu et al., 2012). *In vitro*, studies demonstrate that AGEs significantly enhance platelet aggregation induced by ADP and serotonin through oxidative stress generation and subsequent prostanoid production. Both food- and serum-derived AGEs stimulate CD62 expression (up to 7.1-fold) and CD63 expression (up to 2.2-fold) on platelet membranes in a concentration- and time-dependent manner (Gawlowski et al., 2009; Hasegawa et al., 2002; Taguchi and Fukami, 2023; Zhu et al., 2012). Moreover, AGEs-RAGE binding also activates downstream pathways like MAPK/ERK1/2, p38, PI3K/AKT, and NADPH oxidase, amplifying ROS production beyond initial NF-κB signaling. This elevated ROS levels aggravates prostanoid synthesis (e.g., TXA_2_) and contributes to sustained platelet hyperreactivity by impairing NO bioavailability and promoting mitochondrial dysfunction in diabetic conditions (Lin et al., 2025; Taguchi and Fukami, 2023; Yang et al., 2019). Overall, AGEs formation diabetes increases the risk of platelet aggregation, thereby thrombosis.

### 3.3 Oxidative stress

Chronic hyperglycemia induces excessive oxidative stress in diabetic platelets, with significantly increased ROS generation compared to healthy controls (Yamagishi et al., 2001). This oxidative burden manifests through elevated protein modifications, including 3-nitrotyrosine (5.3-fold increase), 4-hydroxynonenal adducts, carbonyl derivatives, and polyubiquitination. The excessive ROS production triggers phosphorylation of p53, which translocates to mitochondria, promoting mitochondrial dysfunction and determining whether platelets exhibit hyperactivity (mild damage) or apoptosis (severe damage). Mitochondrial abnormalities represent a critical pathological feature in diabetic platelets. Studies demonstrate decreased platelet viability, ATP content, and ΔΨM, alongside increased ROS production in both diabetic patients and animal models. The aldose reductase-mediated pathway plays a pivotal role, where hyperglycemia-induced aldose reductase activation generates ROS, leading to p53 phosphorylation (Ser15), mitochondrial Bcl-xL sequestration, and subsequent mitochondrial damage (Pitocco et al., 2013; Tang et al., 2014). Compensatory upregulation of mitochondrial antioxidants such as SOD2 and PRDX3 (2–3-fold) seen in diabetic platelets is evidence of chronic ROS exposure, and it directly correlates with platelet hyperreactivity (Avila et al., 2012). Furthermore, clinical evidence suggests that diabetic platelets often have lower GSH, reduced glutathione peroxidase activity, and impaired capacity to neutralize H_2_O_2_. Further, N-acetylcysteine, a notable antioxidant increases intraplatelet GSH, lowers platelet–monocyte conjugation, and reduces platelet activation in randomized crossover trials, suggesting oxidative stress is a potent contributor to platelet dysfunction (Treweeke et al., 2012).

**FIGURE 1:**
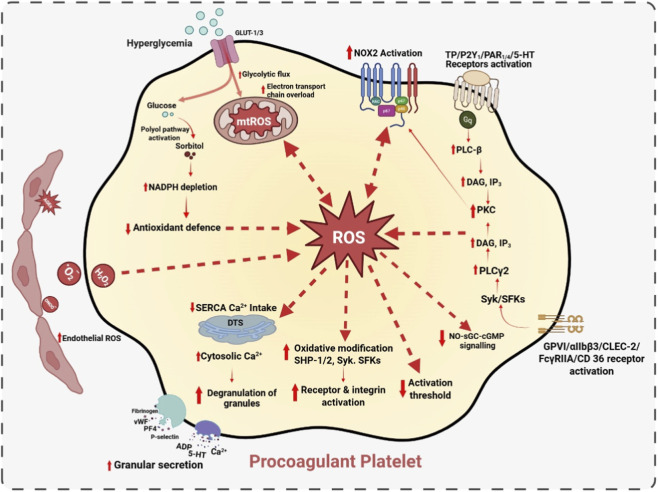
DM-induced ROS signaling in platelets. Hyperglycemia increases platelet glucose uptake via GLUT1/3, enhancing glycolytic flux and mitochondrial electron transport chain activity, leading to elevated mtROS production. Activation of the polyol pathway depletes NADPH and weakens antioxidant defenses, further amplifying oxidative stress. In parallel, hyperglycemia stimulates NOX2 activation and enhances receptor-mediated signaling, promoting PLC-PKC-dependent ROS generation. Accumulated ROS oxidatively modify key signaling proteins, suppress NO-sGC-cGMP inhibitory pathways, impair SERCA-mediated Ca^2+^ reuptake, and elevate cytosolic Ca^2+^. These redox-dependent changes lower the platelet activation threshold, enhance receptor and integrin activation, increase granule secretion, and drive a procoagulant, hyperreactive platelet phenotype under hyperglycemic conditions.

Additionally, oxidative stress directly alters platelet signaling through targeted redox modifications. Mitochondrial superoxide oxidizes critical catalytic cysteine residues in protein tyrosine phosphatases (PTPs), including SHP-2, leading to their functional inactivation (Yamagishi et al., 2001). Since PTPs normally terminate signaling by dephosphorylating kinases, their inhibition results in prolonged phosphorylation of Src family kinases such as Lyn and Fyn, as well as downstream effectors like Syk (Jang et al., 2014). This results in a sustained propagation of activation signals and a lowered threshold for platelet activation. Subsequently, superoxide rapidly reacts with nitric oxide (NO) to generate peroxynitrite, reducing NO bioavailability (Crane et al., 2005). This attenuates activation of sGC and decreases intracellular cGMP levels, thereby weakening inhibitory signaling of the NO-sGC-cGMP pathway that normally suppresses platelet activation. Impairment of the NO-sGC-cGMP pathway removes an essential physiological brake, further reinforcing the hyperactive platelet phenotype under oxidative stress conditions (Crane et al., 2005). Figure 1 summarizes how hyperglycemia increases platelet ROS generation, amplifying signaling and promoting a procoagulant phenotype.

### 3.4 Calcium dysregulation

Mitochondrial Ca^2+^ uptake in platelets, mediated by voltage-dependent anion channels (VDACs) and the MCU complex, plays a central role in regulating platelet activation, bioenergetics, and procoagulant responses (Ajanel et al., 2023; Kholmukhamedov et al., 2018). Controlled Ca^2+^ entry supports energy production, but excessive mitochondrial Ca^2+^ loading can trigger mPTP opening, cytochrome c release, and apoptosis, promoting a prothrombotic state. Conversely, reduced MCU activity limits mitochondrial Ca^2+^ flux, lowering ROS formation, platelet aggregation, and metabolic activation, ultimately producing an antithrombotic phenotype without impairing normal haemostasis. These findings underscore mitochondrial Ca^2+^ handling as a critical determinant of platelet reactivity and a promising target for modulating thrombotic risk (Kholmukhamedov et al., 2018).

In diabetes mellitus, elevated cytosolic Ca^2+^ levels directly impair platelet mitochondrial health by overwhelming the MCU/VDAC uptake system, causing matrix Ca^2+^ overload that opens mPTP pores, collapses ΔΨm, and unleashes ROS/cytochrome c-driven apoptosis alongside prothrombotic hyperactivity (Wang et al., 2025). Moreover, diabetic platelets exhibit profound alterations in Ca^2+^ handling mechanisms. The Na^+^/Ca^2+^ exchanger (NCX) operates in reverse mode in diabetic platelets, mediating Ca^2+^ influx rather than efflux, contributing to elevated intracellular Ca^2+^ and hyperactivity (Roberts et al., 2012). Studies demonstrate that CB-DMB (a NCX inhibitor) decreases Ca^2+^ responses in diabetic platelets but increases them in normal platelets, confirming this directional shift. Additionally, enhanced store-operated Ca^2+^ entry (SOCE) through STIM1 modulates platelet activity by increasing Ca^2+^ influx in diabetic patients with peripheral artery disease (Li et al., 2001; Xia et al., 2015). Persistent partial depletion of intracellular Ca^2+^ stores maintains STIM1 activation and sustains ORAI-1-mediated SOCE, generating a feed-forward amplification loop that prolongs cytosolic Ca^2+^ elevation (Millington-Burgess and Harper, 2021). Critically, altered Ca^2+^ clearance mechanisms of mitochondrial uptake amplify this dysregulation (Dean, 2010). Plasma membrane Ca^2+^-ATPases (PMCA), mainly PMCA1 (ATP2B1) and PMCA4 (ATP2B4) are responsible for restoring basal cytosolic Ca^2+^ levels following platelet activation (Millington-Burgess and Harper, 2021). Moreover, hyperglycemia induced oxidative modifications of P-type ATPase structure further reduce ATP-dependent pump efficiency, leading to delayed efflux of Ca^2+^ ions and prolonged cytosolic Ca^2+^ accumulation (Jardín et al., 2006; Rosado et al., 2004).

Diabetes models show slower platelet Ca^2+^ clearance after activation, matching reduced PMCA function despite unchanged PMCA expression levels (Dean, 2010; Millington-Burgess and Harper, 2021). ATP depletion secondary to mitochondrial dysfunction further limits PMCA turnover, establishing a reciprocal cycle in which impaired extrusion promotes mitochondrial Ca^2+^ overload, which in turn worsens energetic insufficiency and ROS production (Millington-Burgess and Harper, 2021). Similarly, sarco/endoplasmic reticulum Ca^2+^-ATPase (SERCA) dysfunction contributes to sustained Ca^2+^ imbalance. SERCA activity is highly sensitive to oxidative modification, and hyperglycaemic conditions promote cysteine oxidation and glycation of ATP-binding domains, reducing store refilling capacity. Incomplete refilling of the DTS maintains chronic activation of STIM1 and perpetuates SOCE, thereby reinforcing cytosolic Ca^2+^ elevation. Reduced SERCA activity has been demonstrated in diabetic models and is closely associated with exaggerated platelet activation and thrombin generation (Rosado et al., 2004; Dally et al., 2007).

Additional modulatory pathways further destabilize Ca^2+^ homeostasis in diabetes. For example, activation of the RAGE receptors enhances PKCβ signaling, which potentiates phospholipase C activation, IP_3_ production, and intracellular Ca^2+^ release (Sharma et al., 2025). PKC-dependent phosphorylation also increase TRPC6-mediated Ca^2+^ influx, compounding SOCE-driven Ca^2+^ entry into the platelets (Vemana et al., 2015). Moreover, chronic hyperglycemia leads to increased PKC activation via increased *de novo* synthesis of DAG, which activates multiple PKC isoforms in diabetic platelets, particularly PKCβ2 and PKCδ (Geraldes and King, 2010), where upregulation of PKCβ2 has been linked to microvascular complications such as retinopathy and nephropathy, and consequent PKCβ inhibition and insulin treatment have been shown to reduce platelet hyperactivity (Kimura et al., 2001). PKC activation enhances platelet responsiveness to ADP and collagen, by increasing intracellular Ca^2+^ mobilization, promoting granule secretion through disrupting the inhibitory signals from cAMP pathway (Pirags et al., 1996; Rask-Madsen and King, 2005). Moreover, PKC hyperactivation in DM also contributes to increased expression of adhesion molecules like ICAM-1, VCAM-1, leading to a higher risk of atherosclerosis (Lien et al., 2021; Rask-Madsen and King, 2005). Concurrently, diabetic platelets exhibit reduced responsiveness to NO and PGI2 signaling, diminishing cGMP-PKG-mediated suppression of Ca^2+^ mobilization and removing an essential inhibitory brake on activation (Degjoni et al., 2022; Suslova et al., 2014). Lipid membrane remodeling and cholesterol enrichment characteristic of diabetic platelets may further alter channel and pump microdomain organization, modifying the kinetics of ORAI-1, TRPC6, PMCA, and NCX (Bohórquez-Hernández et al., 2017; Rosado et al., 2004; Watala et al., 1998).

**FIGURE 2:**
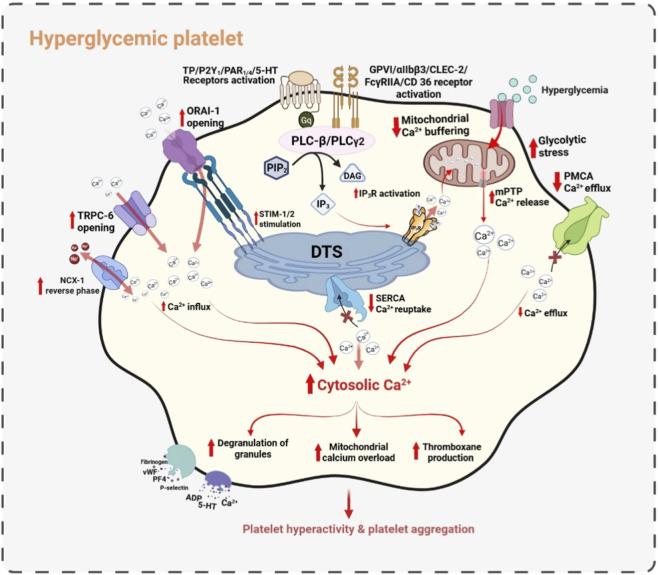
Hyperglycemia-induced Ca^2+^ dysregulation in platelets. Mitochondrial Ca^2+^ buffering is compromised under metabolic stress, contributing to sustained cytosolic Ca^2+^ elevation. Store-operated Ca^2+^ entry via STIM1-ORAI-1, along with TRPC6 and reverse-mode NCX1, further augments Ca^2+^ influx. Simultaneously, impaired SERCA-mediated reuptake and reduced PMCA-dependent efflux limit Ca^2+^ clearance. Hyperglycemia enhances platelet receptor signaling, activating PLC pathways and increasing IP_3_-mediated Ca^2+^ release from the DTS. The resulting Ca^2+^ overload drives granule secretion, thromboxane production, platelet hyperreactivity, and aggregation in hyperglycemic conditions.

Overall, these findings indicate that mitochondrial Ca^2+^ overload in diabetic platelets is not an isolated defect but the downstream consequence of coordinated dysregulation across influx pathways (SOCE, TRPC6, reverse-mode NCX), clearance systems (PMCA and SERCA impairment), redox-sensitive signaling axes (RAGE-PKC), and inhibitory pathways (cAMP and NO-sGC-cGMP). The sustained elevation of cytosolic Ca^2+^ prolongs mitochondrial Ca^2+^ uptake, increases ROS production, lowers the mPTP opening threshold, and drives the transition toward a procoagulant, apoptosis-prone platelet phenotype. Therefore, targeting mitochondrial Ca^2+^ handling is of great therapeutic potential. However, therapeutic efficacy is likely depend on simultaneous modulation of upstream Ca^2+^ extrusion and store-refilling mechanisms to restore global Ca^2+^ homeostasis and reduce thrombotic risk in diabetes. The detailed hyperglycemia-driven Ca^2+^ dysregulation in platelets are depicted in Figure 2.

### 3.5 Platelet mitochondrial dysfunction

Platelets contain a low number of mitochondria and plays a significant role in platelet activation, aggregation, and platelet apoptosis (Ajanel et al., 2023). Early investigations into platelet mitochondrial function in diabetes revealed significant metabolic abnormalities. Yamagishi et al. (2001) first demonstrated that hyperglycaemia promotes mitochondrial superoxide generation in human platelets. When collagen-stimulated hyperglycaemic platelets were treated with the complex II inhibitor, thenoyltrifluoroacetone or the uncoupler CCCP, mitochondrial superoxide levels declined through modulation of the tyrosine kinase Syk. These findings established that mitochondrial ROS acts as a central driver of platelet dysfunction in diabetes (Yamagishi et al., 2001). Further, Ran et al. (2009) reported that platelets from diabetic patients exhibit increased mitochondrial ATP production and reduced ΔΨM, suggesting early compensatory hyperactivation of mitochondrial metabolism. However, later evidence challenged this pattern (Ran et al., 2009). Avila et al. (2012) observed that diabetic platelets actually display reduced oxygen consumption rate (OCR) and impaired oxygen-dependent ATP synthesis, accompanied by increased carbonylation of antioxidant proteins (superoxide dismutase (SOD) and thioredoxin-dependent peroxidase 3), indicating oxidative damage within platelet mitochondria (Avila et al., 2012). Consistent with declining mitochondrial efficiency, Wu et al. (2015) found reduced total and mitochondrial ATP content in platelets from diabetic humans and rats, alongside increased ROS and altered ΔΨM levels, supporting mitochondrial energetic failure in established diabetes (Wu et al., 2015).

In circulating platelets of diabetic rats, mitochondrial mass, ΔΨM, and mitochondrial respiration were increased, suggesting that chronic diabetes promotes mitochondrial biogenesis that may contribute to hyperpolarization-driven platelet hyperreactivity (Siewiera et al., 2016). Surprisingly, mitochondrial genomic alterations may not fully account for these dysfunctions. Karimova et al. (2018) found that the common 4,977-bp mtDNA deletion and mtDNA copy number alterations in platelets do not significantly differ between diabetic and non-diabetic individuals, suggesting functional rather than genetic mitochondrial defects (Karimova et al., 2018).

**FIGURE 3:**
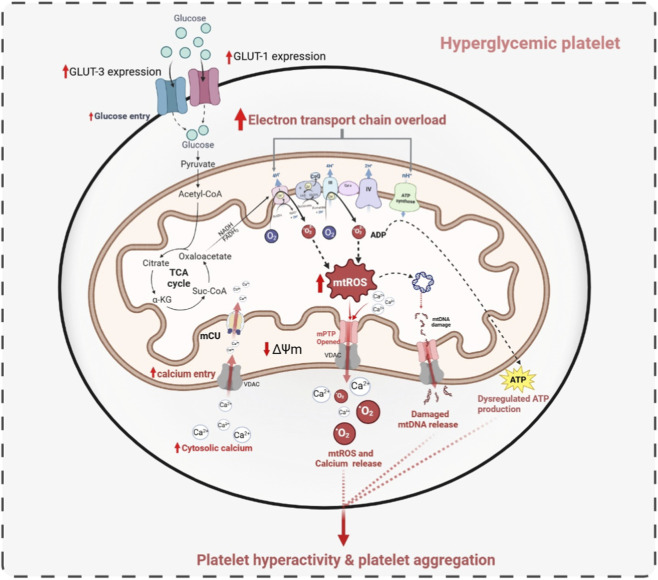
Hyperglycemia increases platelet glucose uptake through GLUT1 and GLUT3, enhancing glycolysis and mitochondrial substrate supply. Elevated NADH and FADH_2_, overloading the electron transport chain and increasing mtROS production. mtROS disrupts ΔΨm, promotes Ca^2+^ overload, and facilitate mPTP opening. These changes impair redox balance and mitochondrial function, leading to the release of mtROS and damaged mitochondrial components. Collectively, these alterations enhance platelet activation, hyperreactivity, and aggregation under hyperglycemic conditions.

Recent findings strengthen the role of electron transport enzymes and OXPHOS defects in platelet mitochondria. Wang et al. (2024) reported that T2DM platelets show complex III deficiency, impaired mitochondrial respiration, increased α-granule activity, and heavy reliance on fatty acid oxidation (FAO). Pharmacological FAO inhibition using trimetazidine, alone or combined with mitochondrial ROS modulators (S3QEL2/SLQEL.1), restored mitochondrial respiration and redox balance (Wang et al., 2024). Prediabetes often progresses to diabetes, and patients and animals with prediabetic profiles exhibit increased platelet aggregation and thrombosis tendencies (Neergaard-Petersen et al., 2015; Shukla et al., 2008). Ahmed et al. showed that accumulation of mitochondrial oxidants promotes platelet activation and thrombosis, which can be reversed by antioxidants such as SOD and mitoQ, indicating that platelet hyperreactivity and thrombosis risk begin early in metabolic dysregulation (Ahmed et al., 2024). Most recently, Agbas et al. (2025) demonstrated that T2DM platelets exhibit a significant decline in non-mitochondrial OCR, likely due to reduced activities of NADPH oxidase, COX, and LOX. Despite modest changes in ATP-linked OCR, reduced non-mitochondrial OCR independently contributes to diabetic peripheral neuropathy, highlighting additional bioenergetic defects beyond mitochondria (Agbas et al., 2025). Like diabetes, platelet mitochondrial dysfunction is reported to be involved in chronic kidney damage in diabetes patients. It has been observed that platelet mitochondrial complex I, II, and OCR significantly declined in diabetic patients with or without chronic kidney disease, indicating that platelet mitochondrial OCR can be considered as a peripheral biomarker for kidney mitochondrial dysfunction (Mihaela-Roxana et al., 2025). Human mut-T Homologue 1 (mTH) is reported to be expressed on platelet mitochondria, and its deficiency could reduce platelet aggregation, Ca^2+^ mobilization, and phosphatidyl serine exposure against thrombin exposure. Further, mTH one deficiency shows mitochondrial DNA oxidative damage when reduced expression of cytochrome C oxidase 1, indicating that targeting mTH one could be a potential strategy to prevent thrombotic events (Ding et al., 2023). Figure 3 depicts the molecular mechanism of platelet hyperactivation in DM.

Parkin is a key regulator of mitochondrial quality control via PINK1/Parkin-mediated mitophagy (Lee et al., 2020). PRKN gene product (Parkin) is an E3 ubiquitin ligase highly expressed in platelets of both healthy individuals and diabetic patients. Physiologically, Parkin maintains mitochondrial number and function by selectively removing damaged mitochondria through mitophagy, thereby sustaining ATP production, which consequently supports normal platelet activation and aggregation during vascular injury (Lee et al., 2016).

Following platelet activation upon vascular injury, reduced ΔΨM allows PINK1 accumulation on the outer mitochondrial membrane (OMM), followed by Parkin recruitment, promoting mitophagy via ubiquitination and autophagosomal degradation. Mechanistically, Parkin ubiquitinates OMM proteins, including mitofusin (Mfn), Mfn1, and Miro, which subsequently recruit LC3 to facilitate mitochondrial fragmentation and autophagosomal clearance, likely involving DRP1-mediated fission. This reduces ROS generation, stabilizes Ca^2+^ signaling, suppresses mtDNA release, and prevents premature apoptosis, which ensures controlled activation and aggregation during vascular injury without hyperactivity (Lee et al., 2020; Lee et al., 2016).

Oppositley, in diabetic conditions, chronic hyperglycemia-induced oxidative stress overwhelms this protective mitophagy response by exceeding the adaptive capacity of Parkin activation, leading to the accumulation of dysfunctional mitochondria in platelets with high ROS-generating potential (Lee et al., 2020). Proteomic analyses have identified numerous parkin-associated proteins in diabetic platelets, including integrin-related and signaling molecules such as protein disulfide isomerase 1 (PDI1), integrin-linked kinase (ILK), and fermitin family member 3 (FERMT3), and 14-three to three, suggesting parkin may influence platelet activation pathways (Lee et al., 2020), Moreover, Hyperglycemia-induced mtROS exacerbates mitophagy dysregulation, promoting platelet hyperactivity alongside PKC activation and Ca^2+^ influx, thereby contributing to platelet hyperreactivity and increased thrombotic risk observed in diabetes (Gresele et al., 2010). Collectively, these findings suggest that therapeutic strategies aimed at restoring or enhancing parkin-mediated mitophagy may represent a novel approach to attenuate abnormal platelet activation and thrombosis risk (Lee et al., 2016).

Cumulatively, diabetes-induced mitochondrial dysfunction drives platelet hyperreactivity through oxidative stress, impaired respiration, altered ATP production, and disrupted redox balance. Evidence shows compensatory metabolic shifts, defective mitophagy, and drug-dependent mitochondrial modulation collectively promoting thrombotic risk. Overall, platelet mitochondrial integrity emerges as a pivotal therapeutic target to normalize platelet function in diabetes.

### 3.6 Interplay of calcium, mitochondria, and ROS in platelets in health and diabetes

Ca^2+^ is the central intracellular second messenger that integrates receptor-mediated signaling with secretion, integrin activation, cytoskeletal remodeling, and metabolic adaptation in platelets. Under resting conditions, cytosolic Ca^2+^ is maintained at ∼100 nM through coordinated action of the dense tubular system (DTS), sarco/endoplasmic reticulum Ca^2+^-ATPase (SERCA2b), plasma membrane Ca^2+^-ATPase (PMCA), and the Na^+^/Ca^2+^ exchanger (NCX) (Dolan and Diamond, 2014; Varga-Szabo et al., 2009). Upon platelet agonist stimulation, PLC activation generates IP_3_, triggering rapid Ca^2+^ release from the DTS via IP_3_ receptors. Store depletion activates stromal interaction molecule 1 (STIM1) and Orai1 channels, initiating store-operated Ca^2+^ entry (SOCE). The resulting Ca^2+^ spikes determine the magnitude and duration of platelet functional responses (Lang et al., 2013).

Mitochondria are the critical regulators of Ca^2+^ dynamics. They are positioned near Ca^2+^ release microdomains to sequester cytosolic Ca^2+^ through the MCU, driven by the negative ΔΨm (Shehwar et al., 2025). This uptake buffers cytosolic Ca^2+^ and prevents excessive or sustained elevations. Simultaneously, matrix Ca^2+^ activates key tricarboxylic acid (TCA) cycle dehydrogenases, increasing NADH and FADH_2_ production and enhancing electron transport chain (ETC.) flux and OXPHOS. Thus, Ca^2+^ serves as a stimulating signal that matches mitochondrial ATP generation to activation-induced energy demand. Efflux via the mitochondrial Na^+^/Ca^2+^ exchanger (NCLX) restores matrix Ca^2+^ levels, maintaining redox balance and preserving respiratory reserve capacity (Ajanel et al., 2023; Shehwar et al., 2025).

**FIGURE 4:**
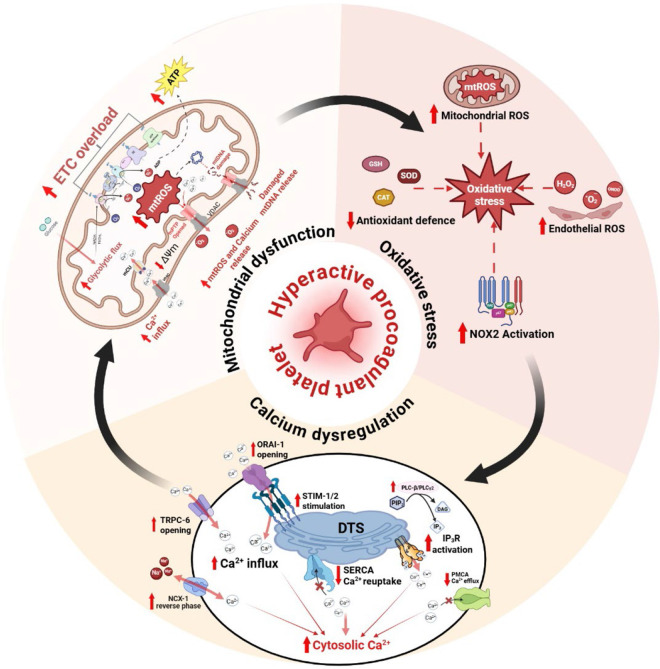
Interplay between mitochondrial dysfunction, oxidative stress, and Ca^2+^ dysregulation in hyperglycemia-induced platelet hyperreactivity. Chronic hyperglycemia induces excessive metabolic flux through platelet mitochondria, leading to ETC, overload, elevated mtROS generation, dysregulated ATP production, loss of ΔΨm leading to sustained mPTP-VDAC mediated release of mtROS and Ca2+. This released mtROS amplifies oxidative stress through NOX2 activation, impairs antioxidant defenses, further enhancing redox-sensitive signaling pathways, while mitigating the inhibitory NO-sGC-cGMP pathways. Oxidative modifications of Ca^2+^-handling proteins promote increased Ca^2+^ influx (via ORAI1/TRPC6), elevated PLC activity induces IP_3_ receptor-mediated Ca^2+^ release, reduced SERCA-dependent reuptake, and diminished PMCA-mediated efflux, resulting in sustained cytosolic Ca^2+^ elevation. Elevated cytosolic Ca^2+^ further increases mitochondrial Ca^2+^ uptake, exacerbating mitochondrial dysfunction and ROS production. These three interconnected processes form a self-reinforcing pathological cycle that lowers the activation threshold of platelets, promotes granule secretion and integrin activation, and sustains a procoagulant phenotype in hyperglycemic conditions.

Mitochondrial health is therefore tightly coupled to Ca^2+^ homeostasis. Under physiological conditions, transient Ca^2+^ elevations promote adaptive bioenergetic responses without destabilizing ΔΨm. However, sustained cytosolic Ca^2+^ elevations can exceed mitochondrial buffering capacity, leading to matrix Ca^2+^ overload, enhanced electron leak at complexes I and III, and increased mitochondrial ROS (mtROS) generation (Choo et al., 2012). Excess ROS and higher baseline ROS in metabolic stress conditions like diabetes further sensitize mPTP, and prolonged pore opening results in ΔΨm dissipation and impaired ATP synthesis (Avila et al., 2012; Lonobile et al., 2025). Overall, both Ca^2+^ and mitochondria coordinated with each other, thereby regulating the platelet function in healthy conditions. Indeed, in diabetes, these triod connections are altered resulting in platelet activation, aggregation, thrombosis, and platelet apoptosis. The detailed mechanism of how this interplay between Ca^2+^, mitochondria, and ROS regulates the platelet activities are illustrated in Figure 4.

## 4 Platelet dysfunction management in diabetes

### 4.1 Antidiabetic agents as antiplatelets

Platelet dysfunction has a significant contribution to cardiovascular risks in diabetic patients. Current antiplatelet and antithrombotic agents shown limited efficacy in reducing the thrombotic risks in diabetic patients due to i) platelet hypersensitivity, ii) haemorrhagic risk, and iii) comorbid diseases. Antidiabetic agents have been reported to exhibit antiplatelet and antithrombotic properties in addition to their glycemic control effects (Nusca et al., 2021). For example, metformin has been shown to reduce both collagen- and arachidonic acid-induced platelet aggregation in preclinical studies (Gin et al., 1989; Tremoli et al., 1982). Additionally, metformin decreases platelet superoxide production and the release of extracellular mitochondrial DNA, which are known to contribute to platelet hyperreactivity and vascular damage (Gargiulo et al., 2002; Xin et al., 2016). Clinically, treatment with metformin significantly lowers 11-dehydro-thromboxane B_2_ (11-dhTxB_2_) levels and mean platelet volume (MPV), indicating a reduction in platelet activation (Dolasık et al., 2013; Formoso et al., 2008).

Sulfonylureas have demonstrated a reduction in ADP-induced platelet adhesiveness and platelet aggregation in preclinical studies (Klaff et al., 1981; Satoh et al., 1994). These effects appear to be partially mediated by decreased oxidative stress and inhibition of the COX and LOX pathways (Satoh et al., 1994; Violi et al., 1982). However, despite their antiplatelet activity, large clinical studies have reported an increased risk of cardiovascular hospitalization, stroke, and mortality, especially when sulfonylureas are combined with metformin (King et al., 1999; Monami et al., 2013; Rao et al., 2008).Thiazolidinediones/glitazones significantly reduced the ADP-induced platelet aggregation and P-selectin expression (Bodary et al., 2005; Li et al., 2005). Clinically, these agents are associated with a reduction in macrophage recruitment, inflammation, E-selectin, vWF, soluble CD40 ligand (sCD40L), PAI-1 and 11-dhTxB_2_ levels, reflecting a marked suppression of platelet-mediated inflammatory and prothrombotic activity (Derosa et al., 2005; Hetzel et al., 2005; Sidhu et al., 2003). In addition, they are reported to decrease platelet mitochondrial respiration, further reducing platelet reactivity (Li et al., 2020).

Sodium-glucose cotransporter 2 (SGLT-2) inhibitors also displayed significant antiplatelet properties. In preclinical studies, these agents inhibited ADP-induced platelet aggregation, ROS production, AGEs, and P-selectin expression, while enhancing endothelial nitric oxide synthase (eNOS) activity and NO bioavailability (Aroor et al., 2018; Smyth et al., 2017; Spigoni et al., 2020; Steven et al., 2017). Clinically, they reduce intracellular free Ca^2+^ levels and platelet tyrosine phosphorylation, contributing to diminished platelet activation (Gupta et al., 2012). Glucagon-like peptide-1 (GLP-1) receptor agonists have also been shown to inhibit ADP, thrombin, and collagen-mediated platelet aggregation, primarily through cAMP-mediated PKA activation, increased endothelial nitric oxide synthase (eNOS) activity (Cameron-Vendrig et al., 2016; Steven et al., 2017) and reduces oxidative stress (Ceriello et al., 2013; Oeseburg et al., 2010). Clinically, GLP-1 agonists increase cyclic GMP (cGMP) production, promote VASP (Ser239) phosphorylation, enhance NO bioavailability, and reduce fibrinogen levels, P-selectin expression, and platelet-monocyte aggregate formation (Barale et al., 2019; Kahal et al., 2015). Although these antidiabetic agents inhibit multiple molecular pathways involved in platelet activation in both preclinical and clinical settings, they fail to completely abolish the persistent platelet dysfunction observed in diabetic patients, which continues to contribute to increased cardiovascular and cerebrovascular risk. These findings highlight the urgent need for future therapies that can simultaneously improve glycemic control and restore normal platelet function in diabetic populations. The antidiabetics and their effects on platelet dysfunction are reported in Table 3.

**TABLE 3:** Effects of antidiabetic agents on platelet dysfunction.

Antidiabetics	Key antiplatelet effects		Mechanism	Limitations	Reference(s)
	Preclinical	Clinical			
Metformin	↓ collagen/AA-induced aggregation ↓ superoxide ↓ extracellular mtDNA	↓ 11-dhTxB_2_ ↓ MPV	↓ oxidative stress ↓ TXA_2_	Does not fully normalize platelet hyperreactivity	Dolasık et al. (2013), Formoso et al. (2008), Gargiulo et al. (2002), Gin et al. (1989), Tremoli et al. (1982), Xin et al. (2016)
Sulfonylureas	↓ ADP-induced adhesiveness and aggregation	-	↓ oxidative stress ↓ COX/LOX signaling	↑ Cardiovascular hospitalization, stroke and mortality risk, especially in combination with metformin	King et al. (1999), Klaff et al. (1981), Monami et al. (2013), Rao et al. (2008), Satoh et al. (1994), Violi et al. (1982)
Thiazolidinediones (glitazones)	↓ ADP-induced aggregation ↓ P-selectin ↓ mitochondrial respiration	↓ inflammation; ↓ E-selectin, vWF, sCD40L, PAI-1, 11-dhTxB_2_	Anti-inflammatory effects ↓ activation markers	Fluid retention and other non-platelet safety issues	Bodary et al. (2005), Derosa et al. (2005), Hetzel et al. (2005), Li et al. (2005), Li et al. (2020), Sidhu et al. (2003)
SGLT-2 inhibitors	↓ ADP-induced aggregation ↓ ROS/AGEs/P-selectin ↑ eNOS → ↑ NO	↓ intracellular Ca^2+^, ↓ tyrosine phosphorylation	↑ NO signaling ↓ oxidative stress, ↓ Ca^2+^-dependent activation	Partial antiplatelet effect in diabetes	Aroor et al. (2018), Gupta et al. (2012), Smyth et al. (2017), Spigoni et al. (2020), Steven et al. (2017)
GLP-1 receptor agonists	↓ ADP/thrombin/collagen-induced aggregation ↓ oxidative stress	↑ cGMP ↑ VASP-P (Ser239) ↑ NO ↓ fibrinogen P-selectin, platelet–monocyte aggregates	↑ cAMP–PKA ↑ eNOS/NO, ↓ platelet–leukocyte interactions	Residual platelet dysfunction persists	Barale et al. (2019), Cameron-Vendrig et al. (2016), Ceriello et al. (2013), Kahal et al. (2015), Oeseburg et al. (2010)

### 4.2 Management of platelet mitochondria

#### 4.2.1 Antidiabetics and antiplatelets

Metformin is well known to suppress mitochondrial complex I activity. Several studies have demonstrated that metformin inhibits platelet aggregation. However, it has been noted that metformin dose dependently enhanced the platelet lactate production and reduced the complex I and IV activities, ΔΨM, and OCR, indicating that metformin-induced mitochondrial toxicity is dose dependent (Piel et al., 2015; Protti et al., 2012). Indeed, all these abnormal platelet mitochondrial activities are linked to promoting platelet aggregation. Although metformin generally inhibits platelet aggregation, these results revealed a dose-dependent mitochondrial toxicity that may paradoxically enhance platelet activation, warranting further clarification (Protti et al., 2012). Complementing this, Xin et al. (2016) showed that metformin protects platelet mitochondria by inhibiting complex I activity, thereby reducing mitochondrial hyperpolarization, ROS generation, and mtDNA release, and consequently preventing mtDNA-mediated platelet activation via the dendritic cell-specific intercellular adhesion molecule-3-grabbing non-integrin (DC-SIGN)-dependent pathway (Xin et al., 2016).

Linagliptin, a DPP-4 inhibitor, reduced the thrombin-induced aggregation through blocking phosphodiesterase activity and enhancing cAMP. In response to thrombin-treated platelets, linagliptin pre-incubation decreased the OCR, altered the structure of activated platelets, and lowered the number and α-granules secretion (Li et al., 2020). In diabetic patients treated with atorvastatin or rosuvastatin, no significant alterations in platelet mitochondrial respiration were observed. However, subsequent treatment with membrane-permeable succinate enhanced platelet mitochondrial bioenergetics in these patients (Avram et al., 2021).

Experimental studies revealed that the commonly used antiplatelet drug aspirin reduces mtROS in the pancreas and heart of diabetic rats through COX-1 inhibition and indirect antioxidant mechanisms (John et al., 2021). However, the role of aspirin in reducing the)Early investigations into platelet mitochondrial function in diabetes revealed significant metabolic abnormalities. Yamagishi et al. (2001) first demonstrated that hyperglycaemia promotes mitochondrial superoxiplatelet mtROS and mitochondrial dysfunction in platelet is still needs to be explored. Moreover, P2Y12 receptor antagonist cangrelor, combined with metabolism inhibitors, reduced mitochondrial respiratory rate and showed enhanced antiplatelet aggregatory activity (Dionisio et al., 2025; Kurpinska et al., 2025). GPIIb/IIIa inhibitors (e.g., tirofiban) improve platelet redox balance by blocking integrin-mediated signaling, preventing ROS amplification from NOX2 and thiol oxidation during aggregation (Dionisio et al., 2025). These mitochondrial-targeted effects suggest that antiplatelets indirectly restore platelet function in diabetes. Although limited research has investigated the antidiabetic agents and antiplatelet-mediated modulation of platelet mitochondria in preventing platelet activation, aggregation, and thrombosis, future research needs to investigate the in-depth mechanism of platelet mitochondria’s role in preventing the thrombotic risks in diabetes.

#### 4.2.2 Antioxidants

Oxidative stress can induce mitochondrial damage and is a key contributor to thrombotic events in diabetes (Giacco and Brownlee, 2010). Therefore, reduction of oxidative stress is not only beneficial to maintain mitochondrial health but also reduces platelet dysfunction in diabetes. Mitochondrial antioxidants like SOD, Mitoquinone (MitoQ), Skulachev’s quinone 1 (SKQ1), and coenzyme Q_1_ (CoQ1) reduced the mtROS and improved the ΔΨM in platelet mitochondria (Sacks et al., 2021). Notably, mitophagy-mediated clearance of damaged mitochondria has been shown to reduce phosphorylated p53 levels, apoptosis, and thereby normalise platelet function. Further induction of mitophagy in platelets also reduced the oxidative stress (Lee et al., 2016). Lifestyle interventions such as diet and physical activity play a crucial role in the long-term management of glucose in diabetes patients and have been shown to reduce the mtROS and improve the mitochondrial biogenesis in diabetes. However, the specific impact of these interventions on platelet mitochondrial function remains largely unexplored and needs further investigation.

#### 4.2.3 Platelet-derived mitochondrial transplantation

Apart from the platelet activation and aggregation roles by platelet mitochondria, platelet-derived mitochondria have been investigated for their potential to improve pathological outcomes in diabetes. For example, platelet-derived mitochondrial transplantation enhances embryonic stem cell markers and enhances beta cell function in diabetics. This could be due to the modulation of immune cell proliferation and function (Zhao et al., 2017). Further platelet-derived mitochondria treatment for 1 month enhanced brain mitochondrial number, reduced oxidative stress, improved mitochondrial function, and reduced apoptosis, which are accompanied by enhanced memory (Ma et al., 2020). These findings suggest that transplantation of platelet mitochondria could be a promising approach in combating diabetes associated complications. Future studies should explore optimized delivery, targeting efficiency, long-term safety, and immunometabolic effects of platelet-derived mitochondrial transplantation. Determining its impact on organ-specific complications, β-cell preservation, neuroprotection, and vascular repair could establish this approach as a novel regenerative therapy for diabetes and its progressive complications.

#### 4.2.4 Mitophagy as a protective response in platelets

Mitochondrial dysfunction is a key contributor to platelet hypersensitivity, irregular activation, and increased aggregation. Accordingly, the removal of dysfunctional mitochondria is considered a potential therapeutic strategy for managing platelet dysfunction. Mitophagy is the principal endogenous quality control pathway that selectively eliminates dysfunctional mitochondria, thereby preserving mitochondrial integrity and maintaining cellular homeostasis (Lee et al., 2016). Notably, mitophagy-mediated clearance of damaged mitochondria has been shown to reduce phosphorylated p53 levels, apoptosis, oxidative stress, and thereby normalize platelet function (Lee et al., 2016), suggesting that induction of mitophagy may have therapeutic potential in limiting platelet hyperactivity and reducing thrombotic risk. Similarly, BCL2/adenovirus E1B 19 kDa protein-interacting protein 3-like (BNIP3L/NIX)-driven mitophagy maintains platelet mitochondrial quality, supports activation, regulates lifespan, and modulates arterial thrombosis, offering a promising antithrombotic therapeutic target (Zhang et al., 2019). Parkin recruitment has been shown to induce mitophagy as a protective response by maintaining the mitochondrial number (Lee et al., 2016). Parkin-mediated ubiquitination of mitochondrial outer membrane proteins induces mitochondrial fragmentation and autophagosomal clearance of damaged mitochondria, thereby suppressing the ROS, mtDNA release, and eventually leading to controlled platelet aggregation in vascular injury (Lee et al., 2020; Lee et al., 2016). In contrast, Parkin-induced mitochondria is altered in chronic hyperglycemia, thereby promoting platelet hyperactivity and increased thrombotic risk observed in diabetes (Gresele et al., 2010). Despite the beneficial effects of mitophagy induction in activated platelets, limited research has been conducted on this aspect, which needs to be further investigated. Collectively, these findings highlight mitophagy induction as a novel approach to attenuate abnormal platelet activation and thrombosis risk in diabetes. The pharmacological management of platelet mitochondrial function is summarized in Table 4.

**TABLE 4:** The effects of various interventions on platelet mitochondria and their relationship with platelet activation and aggregation.

Intervention	Drug class	Effects on platelet mitochondria	Impact on platelet activation/Aggregation	Reference(s)
Metformin	Biguanide antidiabetic	Inhibits mitochondrial complex I (±IV); ↓ ΔΨM, OCR; ↑ lactate; mitochondrial stress at high dose, ↓ mtROS, and mtDNA release; excessive mitochondrial	↓ aggregation at therapeutic levels; paradoxical activation with mitochondrial toxicity	Protti et al. (2012), Xin et al. (2016)
Linagliptin (DPP-4 inhibition)	Antidiabetic (DPP-4 inhibitor)	↓ OCR; altered ultrastructure; ↓ α-granule content and secretion	↓ thrombin-induced aggregation	Li et al. (2020)
Statins (atorvastatin, rosuvastatin)	Lipid-lowering	Baseline respiration largely unchanged; mitochondrial capacity remains augmentable (e.g., succinate)	Neutral at baseline	Avram et al. (2021)
Aspirin + GPIIb/IIIa inhibitors	COX-1 inhibitor; integrin antagonists	↓ mtROS; improved redox balance	↓ platelet activation and aggregation	Sacks et al. (2021)
P2Y12 receptor stimulation	Purinergic signaling	↑ mitochondrial respiration	Promotes platelet activation	Sacks et al. (2021)
Mitochondrial antioxidants (MitoQ, SKQ1, CoQ_1_, SOD mimetics)	Targeted antioxidant therapies	↓ mtROS; restore ΔΨM	Attenuate platelet hyperreactivity	Sacks et al. (2021)
Mitophagy (general + BNIP3L/NIX pathway)	Quality-control pathway	Clearance of damaged mitochondria; ↓ oxidative stress; preserved mitochondrial fitness	Normalizes activation; regulates lifespan; ↓ apoptosis	Lee et al. (2016), Zhang et al. (2019)
Lifestyle interventions (diet, exercise)	Non-pharmacological	↓ mtROS; ↑ mitochondrial biogenesis (systemic)	platelet-specific effects are unclear	Barale et al. (2023)

### 4.3 Novel targets/Mechanisms

Recent high-sensitivity proteomic analysis identified SEC61 translocon subunit β (SEC61B) as significantly elevated in diabetic platelets. SEC61B forms a Ca^2+^ leak channel in the endoplasmic reticulum (ER) membrane, and its overexpression leads to increased spontaneous Ca^2+^ release, termed as Ca^2+^ leak from the ER stores into the platelet cytosol (Kong et al., 2025). Elevated cytosolic Ca^2+^ directly elevates platelet activation, aggregation, and secretion, which significantly increases thrombotic risk in diabetes. Experimental evidence suggests that this novel therapeutic target, SEC61 inhibition with anisomycin shown to decrease Ca^2+^ leak and platelet aggregation in experimental models (Kong et al., 2025). Diabetic patients demonstrate elevated levels of the stress protein complex HSPA8/Hsp90/CSK2α, which correlates with enhanced platelet aggregation. Experimental studies suggest that targeting these chaperones, or their associated kinase CSK2α, could be a novel strategy to reduce platelet dysfunction and cardiovascular risk in diabetic patients (Chiva-Blanch et al., 2021; Hernández Vera et al., 2012). Thrombospondin-1 (TSP-1): TSP-1 expression is markedly increased in diabetic blood vessels and platelets. Elevated circulating TSP-1 levels correlate with diabetic vascular complications and serve as predictive biomarkers. TSP-1 contributes to endothelial dysfunction, impaired angiogenesis, and accelerated atherosclerosis through its antiangiogenic and pro-apoptotic effects (Guo et al., 2024). Overall, targeting these novel markers and their interrelations with platelet mitochondrial function should be further studied in the future.

## 5 Conclusion, limitations, and future scope

This review highlights platelets as metabolically active regulators of thromboinflammatory responses in diabetes, extending well beyond their traditional role in hemostasis. Chronic hyperglycemia reprograms platelet metabolism through enhanced glucose uptake, mitochondrial dysfunction, oxidative stress, Ca^2+^ dysregulation, and impaired mitophagy, collectively driving a hyperreactive, procoagulant platelet phenotype. These alterations substantially contribute to the thrombotic and vascular complications observed in diabetic patients. The intricate interplay between metabolic stress and platelet mitochondrial integrity underscores the importance of targeting mitochondrial pathways to mitigate vascular risk. Emerging strategies, including mitochondrial antioxidants, metabolic modulators, and mitophagy regulators, offer potential to restore platelet homeostasis and reduce vascular risk.

Despite these advances, the present understanding of platelet mitochondrial function in DM has some gaps. For example, i) most mechanistic insights are derived from preclinical models, and direct clinical evidence linking platelet mitochondrial modulation to improved cardiovascular outcomes is limited, ii) antidiabetic agents such as metformin exhibit dose- and context-dependent effects on platelet mitochondria, which remain incompletely understood, iii) the effects of metabolic status, drug interactions, and disease stage on platelet mitochondrial function have not been systematically evaluated, iv) translation of mitochondrial-targeted antioxidants and mitochondrial transplantation is further constrained by unresolved issues related to delivery, specificity, safety, and long-term efficacy, v) In parallel, nanomedicine-based approaches are emerging as innovative strategies to target platelet dysfunction with enhanced precision. To overcome such limitations, nanoparticle-based delivery systems can selectively target platelets or platelet mitochondria to deliver antioxidants, metabolic modulators, or antithrombotic agents, thereby improving bioavailability while minimizing systemic bleeding risk (Choudhury et al., 2024b; Raghunathan et al., 2022; Zhang et al., 2025). Such platforms offer promising avenues for personalized, mitochondria-directed therapy in diabetic thromboinflammation.

Future research should prioritize patient-stratified studies integrating platelet metabolomics, proteomics, mitochondrial bioenergetics, and functional assays to define disease-stage–specific platelet signatures. Clinical trials should incorporate platelet mitochondrial endpoints, including respiration, ROS production, and mitophagy markers. Development of platelet-selective mitochondrial modulators and evaluation of lifestyle interventions such as diet and exercise represent low-risk, complementary strategies. Addressing whether platelet mitochondrial dysfunction is causal or adaptive, defining platelet heterogeneity, and establishing mitochondrial biomarkers of thrombotic risk will be critical for advancing precision-based therapies.

Collectively, platelet mitochondria emerge as a central nexus linking metabolic dysregulation to thrombotic risk in diabetes. Targeting mitochondrial dysfunction represents a paradigm shift from broad platelet inhibition toward restoration of metabolic and redox balance, with significant potential to reduce cardiovascular burden in diabetic populations.

## Glossary

11-dhTxB2: 11-dehydro-thromboxane B2
AGEs: Advanced glycation end products
AGs: Alpha granules
AID: Autoimmune disorders
APP: Amyloid precursor protein
ATP: Adenosine triphosphate
Bcl-xL: B-cell lymphoma-extra large
CalDAG-GEFI: Calcium and DAG-GTP exchange factor I
cAMP: Cyclic adenosine monophosphate
cGMP: Cyclic guanosine monophosphate
CB-DMB: Na+/Ca2+ exchange inhibitor
CD: Cluster of differentiation (e.g., CD62, CD63)
CEACAM-1/2: Carcinoembryonic antigen-related cell adhesion molecule 1/2
CLEC-2: C-type lectin-like receptor 2
COX: Cyclooxygenase
CSK2: Casein kinase 2
CXCL4/PF4: Platelet factor 4
CXCL8: C-X-C motif chemokine ligand 8
DAG: Diacylglycerol
DCM: Diabetic cardiomyopathy
DGs: Dense granules
DM: Diabetes mellitus
DRP1: Dynamin-related protein 1
DVT: Deep vein thrombosis
EMMPRIN: Extracellular matrix metalloproteinase inducer
ER: Endoplasmic reticulum
eNOS: endothelial nitric oxide synthase
FAO: Fatty acid oxidation
FcγRIIA: Fc gamma receptor IIA
FERMT3: Fermitin family member 3
GFOGER: Glycine-phenylalanine-hydroxyproline-glycine-glutamate-arginine
GLP-1: Glucagon-like peptide-1
GLUT: Glucose transporter (e.g., GLUT-1, GLUT-3)
GP: Glycoprotein (e.g., GPVI, GPIb-IX-V, GPIIb/IIIa, GPIV)
GPCRs: G protein-coupled receptors
GPIa/IIa: Glycoprotein Ia/IIa (integrin α2β1)
GPIb: Glycoprotein Ib
GPIX: Glycoprotein IX
GPV: Glycoprotein V
Hsp90: Heat shock protein 90
HSPA8: Heat shock protein family A (Hsp70) member 8
IGF-1: Insulin-like growth factor 1
IL: Interleukin (e.g., IL-6)
ILK: Integrin-linked kinase
IP receptor: Prostacyclin receptor
IP3: Inositol 1,4,5-trisphosphate
IRs: Insulin receptors
IRS: Insulin receptor substrate
IS: Ischemic stroke
ITAM: Immunoreceptor tyrosine-based activation motif
ITIM: Immunoreceptor tyrosine-based inhibition motif
kindlin-3: Kindlin-3 integrin activator
LAIR-1: Leukocyte-associated immunoglobulin-like receptor-1
LAMP: Lysosome-associated membrane protein (e.g., LAMP-1, LAMP-2)
LC3: Microtubule-associated protein 1 light chain 3
LOX: Lipoxygenase
Lyn: Lymphocyte-specific protein tyrosine kinase
MAPK: Mitogen-activated protein kinase
MCU: Mitochondrial calcium uniporter
Mfn: Mitofusin
MI: Myocardial infarction
Miro: Mitochondrial Rho GTPase
ΔΨm: Mitochondrial membrane potential
mPTP: Mitochondrial permeability transition pore
MPV: Mean platelet volume
mtDNA: Mitochondrial DNA
mtROS: Mitochondrial reactive oxygen species
NAFLD: Non-alcoholic fatty liver disease
NCX: Na+/Ca2+ exchanger
NF-κB: Nuclear factor-κB
NIX: BNIP3-like
NO: Nitric oxide
OMM: Outer mitochondrial membrane
P2X1: P2X purinergic receptor 1
P2Y1/P2Y12: Purinergic receptors P2Y1/P2Y12
PAC-1: Platelet activation complex-1
PAD: Peripheral artery disease
PAI-1: Plasminogen activator inhibitor-1
PAR: Protease-activated receptor (e.g., PAR1, PAR4)
Parkin: E3 ubiquitin ligase (PRKN gene product)
PDGF: Platelet-derived growth factor
PDI1: Protein disulfide isomerase 1
PECAM-1: Platelet endothelial cell adhesion molecule-1
PF4/CXCL4: Platelet factor 4
PINK1: PTEN-induced kinase 1
PKA: Protein kinase A
PLC: Phospholipase C
PMPs: Platelet-derived microparticles
PRKN: Parkin gene
PS: Phosphatidylserine
p53: Tumor protein p53
RAGE: Receptor for advanced glycation end products
Rap1: Ras-related protein 1
RANTES/CCL5: Regulated on activation, normal T cell expressed and secreted
RIAM: Rap1-GTP-interacting adaptor molecule
ROS: Reactive oxygen species
SGLT-2: Sodium-glucose cotransporter 2
SEC61B: SEC61 translocon subunit B
Siglecs: Sialic acid binding Ig-like lectins
SLP-76: SH2 domain-containing leukocyte protein of 76 kDa
SOCE: Store-operated calcium entry
STIM1: Stromal interaction molecule 1
Syk: Spleen tyrosine kinase
TAF-I: Thrombin-activatable fibrinolysis inhibitor
TF: Tissue factor
TGF: Transforming growth factor
TLR: Toll-like receptor (e.g., TLR2, TLR4)
tPA: Tissue-type plasminogen activator
TP: Thromboxane prostanoid receptor
TSP-1: Thrombospondin-1
TXA_2_/TXB_2_: Thromboxane A_2_/B_2_
vWF: von Willebrand factor
Vav: Guanine nucleotide exchange factor Vav
VDACs: Voltage-dependent anion channels
